# Inflammatory Cytokines and Oxidative Stress Markers in Relation to Colorectal Cancer Risk: A Case–Cohort Study in a Korean Population

**DOI:** 10.3390/cancers18030470

**Published:** 2026-01-30

**Authors:** Eunjung Park, Seungju Baek, Jin-Kyoung Oh, Min Kyung Lim, Eun Young Park

**Affiliations:** 1 Department of Cancer Control and Population Health, National Cancer Center Graduate School of Cancer Science and Policy, Goyang 10408, Republic of Korea; eunjungpark@ncc.re.kr (E.P.); jkoh@ncc.re.kr (J.-K.O.); 2 Department of Public Health, College of Medicine, Korea University, Seoul 02814, Republic of Korea; seungju100@korea.ac.kr; 3 Department of Social and Preventive Medicine, College of Medicine, Inha University, Incheon 22212, Republic of Korea; mickey@inha.ac.kr; 4 Department of Preventive Medicine, College of Medicine, Korea University, Seoul 02814, Republic of Korea

**Keywords:** colorectal cancer, inflammatory cytokines, oxidative stress, interleukin (IL)-6, nitric oxide, obesity, case–cohort study

## Abstract

**Background/Objectives**: Chronic inflammation and oxidative stress are key contributors to colorectal cancer (CRC) development. However, prospective evidence in Asian populations remains limited. This study aimed to investigate the associations between circulating inflammatory cytokines, oxidative markers, and CRC risk in a Korean population. **Methods**: We conducted a case–cohort study nested within the Korean National Cancer Community (KNCCC) Cohort to investigate associations between inflammatory cytokines, oxidative stress markers, and CRC risk. A total of 128 incident CRC cases and 822 subcohort participants were included. Serum levels of interleukin (IL)-6, tumor necrosis factor-α (TNF-α), IL-1β, interferon-γ (IFN-γ), IL-10, reactive oxygen species (ROS), and nitric oxide (NO) were measured. Hazard ratios (HRs) were estimated using the Cox proportional hazards models with Barlow’s weighting. **Results**: Higher serum IL-6 levels were strongly associated with increased CRC risk, with HRs of 6.20 (95% CI: 2.38–16.19), 8.31 (3.24–21.33), and 10.22 (3.95–26.46) for the second through fourth quartiles, compared to the lowest. Detectable levels of IL-1β and IFN-γ were also significantly associated with CRC risk (HRs: 2.16 and 1.53, respectively). Stratified analysis showed that IL-6 and IL-1β were associated with CRC risk in both obese and non-obese participants, while TNF-α, IL-10, and NO were associated with increased risk only among obese individuals. No significant associations were observed for ROS. **Conclusions**: Elevated levels of inflammatory cytokines (IL-6, IL-1β, IFN-γ) and NO were associated with higher CRC risk, suggesting their potential as early biomarkers. Obesity may modify the associations between certain markers and CRC risk. These findings highlight the role of systemic inflammation and oxidative stress in colorectal carcinogenesis.

## Simple Summary

Long-term inflammation and oxidative stress are thought to increase the risk of colorectal cancer (CRC), but evidence from Asian populations is still limited. In this study, we investigated whether blood levels of inflammation-related and oxidative stress-related markers are linked to the future development of CRC in a Korean population. We found that individuals with higher levels of certain inflammatory markers in their blood had a substantially higher risk of developing CRC during follow-up. Some markers were associated with cancer risk regardless of body weight, while others showed stronger associations only among people with obesity, suggesting that excess body fat may influence inflammation-related cancer risk. These results indicate that blood-based markers of inflammation and oxidative stress may help identify people at higher risk of CRC before diagnosis and improve our understanding of how chronic inflammation contributes to cancer development.

## 1. Introduction

Colorectal cancer (CRC) is one of the most common malignancies worldwide and remains a leading cause of cancer mortality [1]. In the Republic of Korea, CRC ranks among the most frequently diagnosed cancers and causes of cancer-related death [2]. Although increased screening has led to declining CRC incidence in older adults, recent data indicate a rising incidence of CRC among younger individuals, highlighting CRC as an emerging public health concern across age groups [3].

CRC development is influenced by a complex interplay of genetic and environmental factors, including diet, obesity, physical inactivity, smoking, and alcohol consumption [4]. Chronic inflammation is thought to be a key biological mechanism linking these risk factors to colorectal carcinogenesis [5]. Persistent inflammatory signaling can promote tumor initiation and progression by altering the intestinal microenvironment, inducing genomic instability, and facilitating malignant transformation.

Among these risk factors, obesity plays an important role in promoting chronic inflammation. Excess adiposity is associated with adipose tissue inflammation characterized by macrophage infiltration and increased secretion of pro-inflammatory cytokines, while obesity-related increases in gut permeability contribute to metabolic endotoxemia and activation of Toll-like receptor-4–mediated inflammatory signaling. Collectively, these processes generate a systemic pro-inflammatory state that may promote colorectal carcinogenesis [6,7].

Chronic inflammatory states associated with obesity are also closely linked to oxidative and nitrosative stress, which represent additional mechanisms underlying CRC development. Reactive oxygen species (ROS) can induce oxidative DNA damage, genomic instability, and aberrant cellular signaling, while nitric oxide (NO), particularly when produced by inducible nitric oxide synthase, contributes to DNA mutations and tumor progression. Sustained inflammatory signaling further exacerbates oxidative stress, reinforcing carcinogenic processes within the colorectal epithelium [8,9].

Within this inflammatory and oxidative milieu, pro-inflammatory cytokines are key downstream mediators of inflammation-driven tumorigenesis. Interleukin-6 (IL-6) promotes CRC development through activation of multiple downstream signaling pathways, among which the Janus kinase/signal transducer and activator of transcription-3 (JAK/STAT3) pathway plays a central role in driving epithelial cell proliferation, survival, and colorectal tumorigenesis [10]. Tumor necrosis factor-α (TNF-α) contributes to tumor progression via sustained activation of nuclear factor-κB (NF-κB), which regulates the expression of genes involved in inflammation, cell survival, and invasion. Interleukin-1β (IL-1β), a key mediator of inflammasome activation, modulates the tumor microenvironment by promoting immune cell recruitment and pro-tumorigenic inflammatory signaling. Interferon-γ (IFN-γ) exhibits complex, context-dependent effects, exerting both anti-tumor immune surveillance and pro-tumorigenic actions under conditions of chronic inflammation. In contrast, interleukin-10 (IL-10) functions as a critical anti-inflammatory cytokine that limits excessive immune activation and may protect against inflammation-driven colorectal tumorigenesis [11,12,13]. These cytokines were selected in the present study because they represent key mediators of both pro- and anti-inflammatory pathways implicated in CRC development.

However, whether pre-diagnostic circulating levels of these inflammatory mediators are associated with future CRC risk remains unclear, as most evidence to date comes from Western populations. Some prospective studies have reported that higher circulating levels of inflammatory cytokines are associated with greater risk of colorectal neoplasia [14], but data in Asian populations are limited. We aimed to address this gap by evaluating the association between baseline serum levels of specific pro- and anti- inflammatory cytokines (IL-6, TNF-α, IL-1β, IFN-γ, IL-10) and related mediators (ROS, NO) and the subsequent risk of CRC. We conducted a case–cohort study within a large prospective cohort in Korea to test the hypothesis that elevated levels of these inflammatory markers are associated with increased risk of CRC.

## 2. Materials and Methods

### 2.1. Study Design and Population

We conducted a case–cohort study within the Korean National Cancer Center Community (KNCCC) Cohort, a community-based prospective cohort in Korea established to identify cancer risk and protective factors [15]. From 1993 to 2010, the KNCCC cohort enrolled 16,304 adults (aged ≥ 30 years) from five regions (Changwon, Chuncheon, Chungju, Sancheong, and Haman). Participants provided baseline data on demographics, lifestyle, and health behaviors, and donated blood and urine samples for biobanking at −70 °C (1993–2008) or −140 °C (2009–2010). Cohort data were linked to the national cancer registry and death records, with follow-up through 31 December 2017.

**Figure 1:**
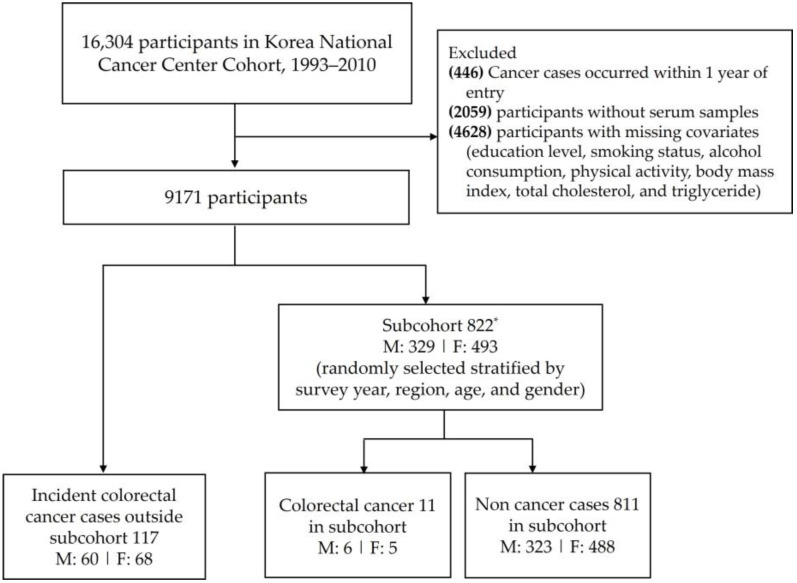
Flowchart of participant selection for the case–cohort study within the Korean National Cancer Center Community (KNCCC) Cohort. A subcohort of 822 participants was randomly selected, stratified by survey year, region, age, and gender. All incident colorectal cancer cases identified through 2017 were included (n = 128), comprising 117 cases outside the subcohort and 11 cases within the subcohort. (M, men; F, women). * The subcohort was randomly selected among participants who entered the cohort between 2001 and 2010.

For this analysis, we excluded individuals with <1 year of follow-up (n = 446), those without an available baseline serum sample (n = 2059), and those missing key covariates (education, smoking, alcohol, or body mass index; n = 4628). From the remaining 9171 eligible participants, we randomly selected a subcohort of 822 participants (stratified by year of entry, region, age, and sex, among those who entered the cohort in 2001–2010). All incident CRC cases in the cohort through 2017 were included (128 cases); 11 of these cases were in the subcohort. Thus, the analysis comprised 128 CRC cases (117 from outside the subcohort + 11 within the subcohort) and 822 subcohort members (including the 11 CRC cases). A flowchart illustrating the selection of participants is shown in Figure 1.

The study protocol was approved by the Institutional Review Board of the National Cancer Center, Korea (IRB No. NCC2020-0203). Written informed consent was obtained from all participants at enrollment, and all procedures were performed in accordance with the Declaration of Helsinki.

### 2.2. Outcome Definition

Incident cancers were identified through registry linkage from 1 January 2001 to 31 December 2017. A first primary CRC was defined by ICD-10 codes C18–C20 (malignancies of the colon, rectosigmoid junction, or rectum). Participants who developed any primary cancer other than CRC prior to a CRC diagnosis were censored at the time of that other cancer diagnosis.

### 2.3. Measurement of Serum Biomarkers

Serum concentrations of five cytokines (IL-6, TNF-α, IL-1β, IFN-γ, and IL-10) were measured using high-sensitivity Quantikine^®^ ELISA kits (R&D Systems, Minneapolis, MN, USA). To ensure analytical reproducibility, all samples were assayed in duplicate, and mean values were used for statistical analyses. The intra-assay and inter-assay coefficients of variation (CVs) were consistently within acceptable ranges for epidemiologic biomarker studies: IL-6 (1.7–4.4% and 2.0–3.7%), TNF-α (4.2–5.2% and 6.8–8.7%), IL-1β (2.8–8.5% and 4.1–8.4%), IFN-γ (2.6–4.7% and 2.7–7.8%), and IL-10 (1.7–5.0% and 5.6–7.6%). Oxidative stress markers, including reactive oxygen species (ROS) and nitric oxide (NO), were quantified using standard colorimetric assays ( BioSource; Thermo Fisher Scientific, Carlsbad, CA, USA and Parameter™; R&D Systems, Minneapolis, MN, USA, respectively). Assay performance was continuously monitored through internal quality-control samples, confirming that all measurements met predefined precision criteria (typically <5% for intra-assay and <10% for inter-assay CVs). Analytical accuracy was further supported by recovery rates ranging from 93% to 107%.

Baseline serum samples were stored at −70 °C (1993–2008) or −140 °C (2009–2010) until analysis. Long-term storage of blood and serum specimens at ultra-low temperatures (−70 °C or below) is recommended to preserve specimen integrity, and potential measurement error related to storage period was addressed by accounting for the year of sample collection in the study design and statistical analyses [16,17]. Values below the LOD were assigned by replacing them with LOD/√2 for statistical analysis.

### 2.4. Statistical Analysis

Follow-up time was calculated from 1 January 2001 (start of cancer follow-up) to the date of first cancer diagnosis, cancer-related death, or end of follow-up (31 December 2017), whichever occurred first. Individuals were censored at the time of any primary cancer diagnosis other than CRC, at death (if not due to cancer), or at the end of follow-up. We compared baseline characteristics of CRC cases and subcohort members, including age, sex, year of cohort entry region, education level (elementary or less, middle school, high school, college or higher), smoking status (never, former, current), alcohol intake (none; moderate ≤ 24 g/day; heavy > 24 g/day), body mass index (BMI, kg/m^2^; analyzed as a continuous variable and categorized as <25 vs. ≥25), physical activity (metabolic equivalent of task [MET]-min/week; low < 600; moderate 600–<3000; high ≥ 3000), meat consumption (almost never, <3 times/week, ≥3 times/week), and vegetable/fruit consumption (≤6 times/week, daily).

Serum cytokine concentrations were log-transformed to improve normality of distributions. Cytokine levels were primarily categorized into quartiles based on their distribution in the subcohort. For cytokines in which >50% of subcohort participants had values below the LOD, the variable was analyzed as binary (LOD vs. >LOD) instead of quartiles. Hazard ratios (HRs) and 95% confidence intervals (CIs) for CRC incidence were estimated using Cox proportional hazards regression with attained age as the time scale [18]. Barlow’s weighted likelihood method was used to account for the case–cohort sampling design [19]. We first fit models adjusted for sex only, and then multivariable models adjusting for sex, year of cohort entry, region, education, smoking, alcohol, BMI, physical activity, meat intake, and vegetable/fruit intake. We also conducted analyses stratified by obesity status (non-obese: BMI < 25; obese: BMI ≥ 25) to evaluate differences in associations by obesity. The proportional hazards assumption was evaluated using Schoenfeld residuals, and no violations were observed. All analyses were performed in SAS version 9.4 (SAS Institute, Cary, NC, USA), and all tests were two-sided with a significance level of 0.05.

## 3. Results

### 3.1. Baseline Characteristics

A total of 950 participants were included in the case–cohort analysis, comprising 128 incident CRC cases and 822 subcohort participants. The mean (SD) age was 63.8 (9.7) years for CRC cases and 60.1 (10.8) years for subcohort members. The median follow-up time was 6.55 years (interquartile range [IQR] 3.86–8.93) for CRC cases and 11.43 years (IQR 8.48–13.88) for the subcohort. This corresponded to 865 person-years of follow-up among CRC cases and 9004 person-years in the subcohort.

CRC cases were, on average, older and had a higher proportion of females compared to subcohort members. Cases also had higher proportions of ever-smokers, alcohol drinkers, individuals with obesity (BMI ≥ 25), and lower educational attainment, and they consumed fruits and vegetables less frequently than subcohort members. Physical activity level and meat intake were similar between the two groups. The detailed sociodemographic and lifestyle characteristics of the participants are presented in Table 1. At baseline, the geometric mean serum concentrations of IL-6, TNF-α, and NO were higher in CRC cases than in subcohort participants (Table 2).

### 3.2. Associations Between Serum Levels of Inflammatory Cytokines and Oxidative Stress and CRC Risk

**Figure 2:**
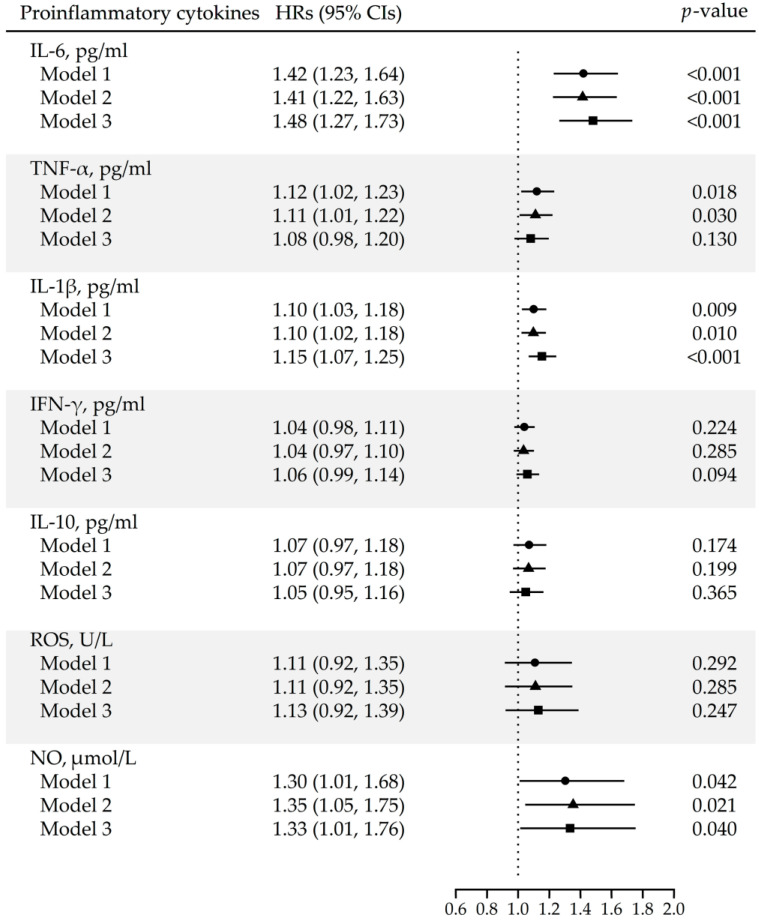
Multivariable-adjusted hazard ratios (HRs) for colorectal cancer risk according to levels of inflammatory cytokines and oxidative stress markers.

Higher circulating levels of pro-inflammatory cytokines were associated with increased risk of developing CRC. In continuous analyses, a one-unit increase in the natural log of a cytokine level (approximately a 2.74-fold increase in concentration) was associated with a multivariable-adjusted hazard ratio (HR) of 1.48 (95% confidence interval [CI]: 1.27–1.73) for IL-6, 1.15 (1.07–1.25) for IL-1β, and 1.33 (1.01–1.76) for NO, as shown in Figure 2. When cytokine levels were categorized into quartiles, the risk of CRC rose markedly with increasing IL-6 concentration. Compared to the lowest quartile of IL-6, the adjusted HRs for the second, third, and fourth quartiles were 6.20 (95% CI: 2.38–16.19), 8.31 (3.24–21.33), and 10.22 (3.95–26.46), respectively (Table 3). Having elevated IL-1β and IFN-γ levels (above the LOD, versus undetectable) was also associated with higher CRC risk (HRs: 2.16 [1.46–3.21] and 1.53 [1.04–2.27], respectively). In contrast, baseline levels of TNF-α, IL-10, and ROS were not significantly associated with CRC risk.

Hazard ratios (HRs) and 95% confidence intervals (CIs) were estimated using Cox proportional hazards models for each inflammatory marker. Three models are shown to demonstrate how the estimated associations change as potential confounding factors are progressively controlled: Model 1, unadjusted; Model 2, adjusted for age and sex, which are fundamental determinants of cancer risk; and Model 3, additionally adjusted for demographic, lifestyle, and health-related factors that may influence both inflammatory marker levels and colorectal cancer risk (i.e., year of cohort entry, region, educational level, smoking status, alcohol consumption, body mass index, physical activity, meat consumption, and vegetable/fruit consumption).

### 3.3. Associations Stratified by Obesity Status

When stratified by obesity status, the associations between inflammatory markers and CRC risk were similar in obese and non-obese groups for IL-6 and IL-1β. Both markers were significantly associated with CRC risk in each group. The adjusted hazard ratio (HR) per one-unit increase in the natural log-transformed IL-6 concentration was 1.66 (95% CI: 1.30–2.12) among obese participants and 1.62 (95% CI: 1.30–2.03) among non-obese participants. Corresponding HRs for IL-1β were 1.21 (95% CI: 1.06–1.39) and 1.13 (95% CI: 1.02–1.26), respectively (Table 4).

In contrast, TNF-α, IL-10, and NO were associated with CRC risk only among obese participants in stratified analyses. Among these biomarkers, statistical evidence of interaction with obesity status was observed only for IL-10 (*p* for interaction = 0.027), whereas no statistically significant interactions were detected for the remaining markers (*p* for interaction: IL-6 = 0.118, TNF-α = 0.521, IL-1β = 0.162, IFN-γ = 0.418, ROS = 0.563, NO = 0.155).

## 4. Discussion

The present study observed a significant association between higher serum levels of pro-inflammatory cytokines (IL-6, IL-1β, IFN-γ) and the inflammatory mediator NO with an increased risk of CRC. These associations remained significant even after multivariable adjustment. Stratified analyses further revealed that IL-6 and IL-1β were associated with CRC risk in both obese and non-obese groups, whereas TNF-α, IL-10, and NO showed associations only in the obese group.

Inflammation plays a critical role in colorectal carcinogenesis [20]. Previous studies have shown that pro-inflammatory cytokines such as IL-6, IL-1β, and TNF-α can influence CRC development and progression. Elevated IL-6, IL-1β, and TNF-α levels have been associated with advanced tumor stage, poorer survival, and metastasis [21]. Notably, IL-6 levels have been shown to correlate with tumor differentiation, suggesting its potential as a diagnostic and prognostic biomarker [22,23,24]. Our findings provide additional evidence that IL-6 and IL-1β are strongly associated with CRC risk, consistent with observations in our earlier studies on lung and gastric cancers in Korean populations [25,26], which suggests common inflammatory pathways across different cancer types.

Extending these observations, our results are broadly consistent with findings from previous studies conducted in both Asian and Western populations. In Asian cohorts, elevated circulating levels of inflammatory markers (IL-6, C-reactive protein (CRP)) have been associated with increased risk of colorectal neoplasia and poorer clinical outcomes, supporting a role for systemic inflammation in colorectal carcinogenesis across diverse Asian populations [27,28]. Similar associations have also been reported in prospective studies from North America, where higher prediagnostic circulating IL-6 levels have been associated with an increased risk of colon cancer [29]. Differences in the magnitude and pattern of these associations, especially those modified by obesity status, may reflect population-specific distributions of adiposity, metabolic profiles, dietary patterns, and genetic backgrounds. The present study contributes to this literature by providing prospective evidence from a Korean cohort and by highlighting obesity as an important biological effect modifier in inflammation-related CRC risk.

IFN-γ, a cytokine involved in cell-mediated immunity, exhibits both anti-tumor and tumor-promoting effects depending on the context. It can inhibit tumor cell growth and activate immune cells for tumor surveillance. However, under certain conditions, IFN-γ may aid tumor survival by promoting immune evasion, anti-apoptotic signaling, and the expansion of immunosuppressive cells [30,31,32,33]. These dual roles of IFN-γ underscore the importance of further investigation into its role in cancer development and therapy. Emerging evidence suggests that the net effect of IFN-γ is highly dependent on the inflammatory and metabolic milieu. While acute IFN-γ signaling supports anti-tumor immunity, chronic or dysregulated IFN-γ exposure may induce immune exhaustion and upregulation of immune checkpoint pathways [34]. Obesity-associated low-grade inflammation may contribute to this shift by sustaining prolonged IFN-γ signaling within a metabolically stressed microenvironment, potentially favoring immunosuppressive and tumor-promoting processes [35]. In our study, the association between IFN-γ and CRC risk observed in overall analyses, despite the absence of a clear obesity-specific pattern, suggests that IFN-γ may reflect heterogeneous immune activation states shaped by complex interactions between systemic inflammation and metabolic context.

TNF-α, IL-10, and NO were associated with CRC risk only among obese individuals in stratified analyses. However, statistical evidence of interaction was observed only for IL-10. For TNF-α and NO, although obesity-specific associations were identified, statistical interaction with obesity was not observed in the present study. Obesity creates a chronic inflammatory state characterized by adipose-derived secretion of cytokines such as IL-6 and TNF-α, and is known to elevate systemic levels of pro-inflammatory mediators [36,37,38,39]. In this context, TNF-α has been shown to activate oncogenic signaling pathways like NF-κB and Wnt/β-catenin, which promote colorectal tumorigenesis [40]. Although TNF-α was associated with CRC risk only among obese participants in stratified analyses, statistical evidence of interaction with obesity was not observed in the present study, despite biological plausibility and prior experimental evidence linking TNF-α-mediated inflammation to obesity-related carcinogenesis.

IL-10, though generally considered anti-inflammatory, may promote tumor progression by suppressing effective immune responses. High IL-10 levels can inhibit cytotoxic T cell activity and enhance regulatory T cell recruitment, creating an immunosuppressive microenvironment that facilitates tumor development [41]. Notably, IL-10 levels are elevated in obesity but paradoxically reduced in severe metabolic dysfunction [42]. In the present study, a statistically significant interaction between IL-10 and obesity was observed, suggesting that the immunoregulatory role of IL-10 may be altered under conditions of chronic inflammation, favoring immune tolerance and colorectal carcinogenesis.

Obesity is also known to increase inducible nitric oxide synthase (iNOS) activity, leading to chronic overproduction of NO [43]. NO and its reactive intermediates can cause oxidative DNA damage, impair apoptosis, and promote angiogenesis and immunosuppression—hallmarks of carcinogenesis [12,44,45,46]. Although NO was associated with CRC risk only among obese participants in stratified analyses, statistical evidence of interaction with obesity was not observed in the present study, despite prior experimental and epidemiologic evidence supporting a role of obesity-related nitrosative stress in colorectal carcinogenesis.

Despite strong experimental and epidemiologic evidence implicating oxidative stress in colorectal carcinogenesis, we did not observe a significant association between circulating ROS levels and CRC risk in this study. Several explanations may account for this null finding. ROS are highly reactive and short-lived molecules; in particular, species such as superoxide anion exhibit extremely short half-lives and are primarily detectable at the site of generation using real-time cellular techniques. Because of these properties, circulating ROS levels are more likely to reflect transient or acute oxidative stress rather than the cumulative, chronic oxidative burden relevant to long-term carcinogenesis. Accordingly, single baseline serum measurements may not adequately capture long-term or tissue-specific oxidative stress within the colorectal epithelium, where carcinogenic processes occur [47,48]. In addition, oxidative stress-related carcinogenesis may be better captured by downstream markers of oxidative damage, such as DNA oxidation or lipid peroxidation products, rather than by direct measurement of circulating ROS [49]. Measurement variability and potential attenuation related to long-term sample storage may have further biased associations toward the null [17]. Therefore, the absence of a significant association likely reflects limitations in exposure assessment rather than the absence of a biological role for oxidative stress in CRC development.

From a clinical perspective, individuals with obesity may represent a subgroup in whom biomarker-based risk assessment could be particularly informative. Biomarkers such as IL-6, TNF-α, and NO may help identify individuals at elevated risk who could benefit from intensified screening, lifestyle modification, or targeted preventive strategies. Future biomarker-guided interventions, including modulation of inflammatory or nitrosative pathways (e.g., TNF-α- or iNOS-related mechanisms), may therefore need to be stratified by obesity status. Several limitations should be acknowledged. First, lifestyle factors were assessed by self-report at baseline, which may introduce recall bias. Second, inflammatory biomarkers were measured at a single time point, precluding assessment of longitudinal changes in inflammatory status. However, single baseline measurements are a widely accepted and validated approach in large-scale prospective cohort studies evaluating long-term disease risk. In this context, any within-person variability over time would most likely result in non-differential misclassification, which tends to bias associations toward the null. Therefore, the observed associations in our study are likely conservative estimates of the true relationships. Importantly, the high analytical reproducibility demonstrated by low intra- and inter-assay variability supports the reliability of the baseline measurements as indicators of inflammatory status at study entry. Third, although a markedly elevated hazard ratio was observed for the highest quartile of IL-6, the number of colorectal cancer cases in the lowest quartile was relatively small, which may have contributed to instability in the categorical estimates. To address this concern, IL-6 was also evaluated on a continuous scale using log-transformed concentrations, and the association with colorectal cancer risk remained strong and statistically significant. This consistency across different modeling approaches suggests that the observed IL-6 association is robust and not solely driven by the choice of categorical cut-points or the small number of cases in the reference group. Nevertheless, given the limited number of cases in certain exposure categories, these findings should be interpreted with caution and warrant confirmation in larger prospective studies with greater statistical power. Fourth, detailed information on chronic inflammatory diseases (e.g., inflammatory bowel disease, rheumatoid arthritis) and medication use that may influence systemic inflammation, such as non-steroidal anti-inflammatory drugs or statins, was not available in this cohort. Consequently, these factors could not be considered as exclusion criteria or included as covariates in the analyses, and residual confounding cannot be ruled out. However, such unmeasured factors are unlikely to be differentially distributed with respect to the outcome and would therefore be expected to attenuate, rather than exaggerate, the observed associations. Finally, because the study population consisted exclusively of Asian participants, the generalizability of the findings to other populations may be limited.

## 5. Conclusions

In conclusion, this study provides evidence that elevated circulating levels of pro-inflammatory cytokines, particularly IL-6 and IL-1β, as well as nitric oxide, are associated with an increased risk of colorectal cancer. These associations remained robust after adjustment for major demographic and lifestyle risk factors.

Overall, these findings support the involvement of systemic inflammation in colorectal carcinogenesis and suggest that circulating inflammatory biomarkers may have potential utility in colorectal cancer risk assessment. Given the substantial global burden of colorectal cancer, an improved understanding of inflammation-related mechanisms may contribute to the development of prevention strategies and approaches for early risk identification. Further prospective studies are warranted to confirm these findings and to clarify the clinical relevance of inflammatory biomarkers across diverse populations.

## Figures and Tables

**Table 1:** Baseline characteristics of case–cohort study subject.

	Colorectal Cancer Cases (n = 128)	Subcohort Participants (n = 822)
Subcohort person-years	865	9004
Age, years, mean ± SD	63.80 ± 9.72	60.11 ± 10.80
Sex, n (%)		
Men	60 (46.88)	329 (40.02)
Women	68 (53.13)	493 (59.98)
Region, n (%)		
San-cheong	44 (34.38)	441 (53.65)
Ui-ryeong	9 (7.03)	27 (3.28)
Chang-won	26 (20.31)	121 (14.72)
Choon-cheon	11 (8.59)	62 (7.54)
Choong-joo	15 (11.72)	76 (9.25)
Ham-an	23 (17.97)	95 (11.56)
Year of entry into the cohort, n (%)		
2001	14 (10.94)	72 (8.76)
2002	6 (4.69)	16 (1.95)
2003	26 (20.31)	107 (13.02)
2004	28 (21.88)	147 (17.88)
2005	21 (16.41)	103 (12.53)
2006	19 (14.84)	129 (15.69)
2008	5 (3.91)	104 (12.65)
2009	6 (4.69)	73 (8.88)
2010	3 (2.34)	71 (8.64)
Educational level, n (%)		
Elementary school or less	37 (28.91)	208 (25.30)
Middle school	73 (57.03)	472 (57.42)
High school	14 (10.94)	109 (13.26)
College or more	4 (3.13)	33 (4.01)
Smoking status, n (%)		
Never-smokers	75 (58.59)	520 (63.26)
Former smokers	25 (19.53)	140 (17.03)
Current smokers	28 (21.88)	162 (19.71)
Alcohol consumption status, n (%)		
Abstainers	63 (49.22)	477 (58.03)
Moderated drinkers	41 (32.03)	211 (25.67)
Heavy drinkers	24 (18.75)	134 (16.30)
Body mass index (BMI), mean ± SD)	24.03 ± 3.18	23.82 ± 3.29
<25 kg/m^2^	78 (60.94)	543 (66.06)
≥25 kg/m^2^	50 (39.06)	279 (33.94)
Physical activity, n (%)		
Low	55 (42.97)	342 (41.61)
Moderate	11 (8.59)	88 (10.71)
High	62 (48.44)	392 (47.69)
Meat consumption, n (%)		
Almost never	48 (37.50)	311 (37.83)
<3 times per week	41 (32.03)	246 (29.93)
≥3 times per week	39 (30.47)	265 (32.24)
Vegetable/fruit consumption, n (%)		
<5 times per week	260 (31.63)	50 (39.06)
≥5 times per week	562 (68.37)	78 (60.94)

SD, standard deviation.

**Table 2:** Serum levels of inflammatory cytokines and oxidative stress stratified by colorectal cancer cases and subcohort.

	Colorectal Cancer Cases							Subcohort							
	GM (95% CI)	Distribution						GM (95% CI)	Distribution						%<LOD
		n	Min	25th	Median	75th	Max		n	Min	25th	Median	75th	Max	
IL-6 (pg/mL)	2.63 (2.13–3.26)	126	LOD	1.89	2.80	4.42	288.24	0.97 (0.84–1.12)	811	LOD	0.82	1.95	3.49	158.33	11.74
TNF-α (pg/mL)	2.25 (1.65–3.07)	124	LOD	2.04	3.74	5.61	13.97	1.22 (1.04–1.44)	816	LOD	1.04	3.23	5.58	25.51	14.57
IL-1β (pg/mL)	0.10 (0.06–0.15)	122	LOD	LOD	0.19	1.02	6.33	0.06 (0.05–0.07)	799	LOD	LOD	LOD	0.68	47.89	53.64
IFN-γ (pg/mL)	0.08 (0.05–0.13)	121	LOD	LOD	0.01	1.57	10.62	0.06 (0.05–0.07)	792	LOD	LOD	LOD	1.39	63.06	61.45
IL-10 (pg/mL)	0.20 (0.14–0.28)	108	LOD	0.09	0.33	0.72	2.49	0.14 (0.12–0.16)	769	LOD	LOD	0.29	0.70	11.61	25.31
ROS (U/L)	895.20 (814.60–983.80)	127	423.40	615.10	770.00	1188.80	4074.40	778.00 (705.90–857.50)	817	LOD	612.90	840.20	1464.20	8761.50	2.44
NO (µmol/L)	60.03 (53.42–67.46)	128	10.15	39.20	53.33	94.00	433.80	51.10 (48.83–53.47)	807	3.63	31.86	49.28	77.31	459.36	0.00

Abbreviations: GM, geometric mean; LOD, limit of detection; IL-6, interleukin 6; TNF-α, tumor necrosis factor-α; IL-1β, interleukin 1β; IFN-γ, interferon gamma; IL-10, interleukin 10; ROS, reactive oxygen species; NO, nitric oxide. LOD: 0.01 pg/mL (IL-6), 0.01 pg/mL (TNF-α), 0.01 pg/mL (IL-1β), 0.01 pg/mL (IFN-γ), 0.01 pg/mL (IL-10), 0.50 U/L (ROS), 0.01 µmol/L (NO).

**Table 3:** Associations between serum levels of inflammatory cytokines and oxidative stress, and CRC risk by quartiles.

				Model 1		Model 2		Model 3	
	Range	Case/ Subcohort	Subcohort Person-Years	HR (95% CI)	*p*-Value	HR (95% CI)	*p*-Value	HR (95% CI)	*p*-Value
IL-6 (pg/mL)									
1st quartile	<0.82	5/203	2391	Reference		Reference		Reference	
2nd quartile	0.82–1.95	30/203	2158	5.95 (2.31, 15.36)	<0.001	5.87 (2.28, 15.15)	<0.001	6.20 (2.38, 16.19)	<0.001
3rd quartile	1.95–3.49	43/204	2191	7.73 (3.05, 19.59)	<0.001	7.48 (2.95, 18.96)	<0.001	8.31 (3.24, 21.33)	<0.001
4th quartile	3.49–158.33	48/201	2135	8.44 (3.34, 21.33)	<0.001	8.38 (3.32, 21.17)	<0.001	10.22 (3.95, 26.46)	<0.001
TNF-α (pg/mL)									
1st quartile	<1.04	21/204	2194	Reference		Reference		Reference	
2nd quartile	1.04–3.23	33/204	2250	1.54 (0.89, 2.65)	0.125	1.46 (0.84, 2.52)	0.181	1.40 (0.79, 2.48)	0.247
3rd quartile	3.23–5.58	38/204	2191	1.65 (0.97, 2.81)	0.068	1.59 (0.93, 2.71)	0.091	1.54 (0.88, 2.71)	0.134
4th quartile	5.58–25.51	32/204	2302	1.35 (0.78, 2.36)	0.286	1.28 (0.74, 2.24)	0.380	1.03 (0.57, 1.88)	0.912
IL-1β (pg/mL)									
<LOD	<0.01	53/441	4912	Reference		Reference		Reference	
≥LOD	≥0.01	69/358	3832	1.66 (1.16, 2.38)	0.005	1.65 (1.16, 2.37)	0.006	2.16 (1.46, 3.21)	<0.001
IFN-γ (pg/mL)									
<LOD	<0.01	67/494	5607	Reference		Reference		Reference	
≥LOD	≥0.01	54/298	3060	1.35 (0.94, 1.94)	0.102	1.33 (0.93, 1.91)	0.124	1.53 (1.04, 2.27)	0.033
IL-10 (pg/mL)									
1st quartile	<0.01	19/203	2113	Reference		Reference		Reference	
2nd quartile	0.01–0.29	30/184	2050	1.58 (0.89, 2.82)	0.118	1.59 (0.89, 2.82)	0.117	1.55 (0.86, 2.78)	0.146
3rd quartile	0.29–0.70	32/190	2119	1.68 (0.95, 2.97)	0.072	1.71 (0.97, 3.01)	0.065	1.45 (0.80, 2.60)	0.218
4th quartile	0.70–11.61	27/192	2131	1.37 (0.76, 2.46)	0.299	1.33 (0.74, 2.39)	0.344	1.29 (0.70, 2.38)	0.423
ROS (U/L)									
1st quartile	<612.90	31/205	2287	Reference		Reference		Reference	
2nd quartile	612.90–840.20	43/204	2129	1.38 (0.87, 2.19)	0.176	1.35 (0.85, 2.15)	0.205	1.48 (0.90, 2.43)	0.121
3rd quartile	840.20–1464.2	32/204	2275	1.13 (0.69, 1.86)	0.623	1.10 (0.67, 1.80)	0.722	1.07 (0.63, 1.81)	0.801
4th quartile	1464.2–8761.5	21/204	2262	0.79 (0.45, 1.38)	0.410	0.79 (0.45, 1.37)	0.401	0.89 (0.50, 1.61)	0.709
NO (µmol/L)									
1st quartile	3.63–31.86	20/202	2104	Reference		Reference		Reference	
2nd quartile	31.86–49.28	34/202	2134	1.61 (0.93, 2.80)	0.090	1.62 (0.93, 2.81)	0.088	1.71 (0.97, 3.02)	0.064
3rd quartile	49.28–77.31	34/202	2236	1.75 (1.00, 3.04)	0.049	1.82 (1.04, 3.17)	0.036	1.86 (1.05, 3.31)	0.035
4th quartile	77.31–459.36	40/201	2351	1.74 (1.02, 2.98)	0.043	1.83 (1.07, 3.14)	0.028	1.76 (1.00, 3.10)	0.052

Model 1 was unadjusted; Model 2 was adjusted for age and sex; Model 3 was further adjusted for year of entry into the cohort, region, educational level, smoking status, alcohol consumption, body mass index, physical activity, meat consumption, and vegetable/fruit consumption. Abbreviations: LOD, limit of detection; IL-6, interleukin 6; TNF-α, tumor necrosis factor-α; IL-1β, interleukin 1β; IFN-γ, interferon gamma; IL-10, interleukin 10; ROS, reactive oxygen species; NO, nitric oxide.

**Table 4:** Colorectal cancer risk for each unit increases in natural log-transformed inflammatory cytokines and oxidative stress stratified with obesity.

		Case/ Subcohort	Subcohort Person-Years	HR (95% CI)	*p*-Value
Obese					
(BMI ≥ 25 kg/m^2^)	IL-6 (pg/mL)	49/277	3096	1.66 (1.30, 2.12)	<0.001
	TNF-α (pg/mL)	48/278	3111	1.22 (1.01, 1.48)	0.041
	IL-1β (pg/mL)	47/272	3037	1.21 (1.06, 1.39)	0.006
	IFN-γ (pg/mL)	46/271	3035	1.10 (0.98, 1.23)	0.112
	IL-10 (pg/mL)	40/263	2953	1.37 (1.09, 1.72)	0.008
	ROS (U/L)	50/279	3126	1.31 (0.83, 2.07)	0.245
	NO (µmol/L)	50/272	3036	1.85 (1.08, 3.15)	0.024
Nonobese					
(BMI < 25 kg/m^2^)	IL-6 (pg/mL)	77/534	5778	1.62 (1.30, 2.03)	<0.001
	TNF-α (pg/mL)	76/538	5825	1.07 (0.94, 1.21)	0.320
	IL-1β (pg/mL)	75/527	5707	1.13 (1.02, 1.26)	0.019
	IFN-γ (pg/mL)	75/521	5633	1.06 (0.97, 1.15)	0.239
	IL-10 (pg/mL)	68/506	5461	0.97 (0.85, 1.10)	0.634
	ROS (U/L)	77/538	5827	1.02 (0.81, 1.29)	0.876
	NO (µmol/L)	78/535	5789	1.12 (0.78, 1.61)	0.533

The hazard ratios (HRs) are presented for each unit increase in natural log-transformed pro-inflammatory cytokines and oxidative stress, derived from Cox proportional-hazards models. They are adjusted for age, sex, year of entry into the cohort, region, educational level, smoking status, alcohol consumption, body mass index, physical activity, meat consumption, and vegetable/fruit consumption. Abbreviations: IL-6, interleukin 6; TNF-α, tumor necrosis factor-α; IL-1β, interleukin 1β; IFN-γ, interferon gamma; IL-10, interleukin 10; ROS, reactive oxygen species; NO, nitric oxide.

## Data Availability

The data presented in this study are available in this article.

## Abbreviations

The following abbreviations are used in this manuscript:

CRC	Colorectal cancer
HR	Hazard ratio
CI	Confidence interval
LOD	Limit of detection
IL-6	Interleukin 6
TNF-α	Tumor necrosis factor-α
IL-1β	Interleukin 1β
IFN-γ	Interferon γ
IL-10	Interleukin 10
ROS	Reactive oxygen species
NO	Nitric oxide

## References

[1] Bray F., Laversanne M., Sung H., Ferlay J., Siegel R.L., Soerjomataram I., Jemal A. (2024). Global cancer statistics 2022: GLOBOCAN estimates of incidence and mortality worldwide for 36 cancers in 185 countries. CA Cancer J. Clin..

[2] International Agency for Research on Cancer Republic of Korea. https://gco.iarc.who.int/media/globocan/factsheets/populations/410-korea-republic-of-fact-sheet.pdf.

[3] National Cancer Information Center Trend Analysis of Cancer Incidence (Korean). https://www.cancer.go.kr/lay1/S1T639C643/contents.do.

[4] Sawicki T., Ruszkowska M., Danielewicz A., Niedźwiedzka E., Arłukowicz T., Przybyłowicz K.E. (2021). A Review of Colorectal Cancer in Terms of Epidemiology, Risk Factors, Development, Symptoms and Diagnosis. Cancers.

[5] Schmitt M., Greten F.R. (2021). The inflammatory pathogenesis of colorectal cancer. Nat. Rev. Immunol..

[6] Tilg H., Moschen A.R. (2006). Adipocytokines: Mediators linking adipose tissue, inflammation and immunity. Nat. Rev. Immunol..

[7] Khandekar M.J., Cohen P., Spiegelman B.M. (2011). Molecular mechanisms of cancer development in obesity. Nat. Rev. Cancer.

[8] Xu W., Liu L.Z., Loizidou M., Ahmed M., Charles I.G. (2002). The role of nitric oxide in cancer. Cell Res..

[9] Reuter S., Gupta S.C., Chaturvedi M.M., Aggarwal B.B. (2010). Oxidative stress, inflammation, and cancer: How are they linked?. Free Radic. Biol. Med..

[10] Grivennikov S., Karin E., Terzic J., Mucida D., Yu G.Y., Vallabhapurapu S., Scheller J., Rose-John S., Cheroutre H., Eckmann L. (2009). IL-6 and Stat3 are required for survival of intestinal epithelial cells and development of colitis-associated cancer. Cancer Cell.

[11] Garner H., de Visser K.E. (2020). Immune crosstalk in cancer progression and metastatic spread: A complex conversation. Nat. Rev. Immunol..

[12] Muthusami S., Ramachandran I.K., Babu K.N., Krishnamoorthy S., Guruswamy A., Queimado L., Chaudhuri G., Ramachandran I. (2021). Role of Inflammation in the Development of Colorectal Cancer. Endocr. Metab. Immune Disord. Drug Targets.

[13] Garcia-Anguita A., Kakourou A., Tsilidis K.K. (2015). Biomarkers of Inflammation and Immune Function and Risk of Colorectal Cancer. Curr. Color. Cancer Rep..

[14] Kim S., Keku T.O., Martin C., Galanko J., Woosley J.T., Schroeder J.C., Satia J.A., Halabi S., Sandler R.S. (2008). Circulating levels of inflammatory cytokines and risk of colorectal adenomas. Cancer Res..

[15] Oh J.K., Lim M.K., Yun E.H., Choi M.H., Hong S.T., Chang S.H., Park S.K., Cho S.I., Kim D.H., Yoo K.Y. (2017). Cohort Profile: Community-based prospective cohort from the National Cancer Center, Korea. Int. J. Epidemiol..

[16] Vaught J.B., Henderson M.K. (2011). Biological sample collection, processing, storage and information management. IARC Sci. Publ..

[17] White E. (2011). Measurement error in biomarkers: Sources, assessment, and impact on studies. IARC Sci. Publ..

[18] Cologne J., Hsu W.L., Abbott R.D., Ohishi W., Grant E.J., Fujiwara S., Cullings H.M. (2012). Proportional hazards regression in epidemiologic follow-up studies: An intuitive consideration of primary time scale. Epidemiology.

[19] Barlow W.E., Ichikawa L., Rosner D., Izumi S. (1999). Analysis of case-cohort designs. J. Clin. Epidemiol..

[20] Li J., Huang L., Zhao H., Yan Y., Lu J. (2020). The Role of Interleukins in Colorectal Cancer. Int. J. Biol. Sci..

[21] Chang P.H., Pan Y.P., Fan C.W., Tseng W.K., Huang J.S., Wu T.H., Chou W.C., Wang C.H., Yeh K.Y. (2016). Pretreatment serum interleukin-1β, interleukin-6, and tumor necrosis factor-α levels predict the progression of colorectal cancer. Cancer Med..

[22] Knüpfer H., Preiss R. (2010). Serum interleukin-6 levels in colorectal cancer patients--a summary of published results. Int. J. Color. Dis..

[23] Vainer N., Dehlendorff C., Johansen J.S. (2018). Systematic literature review of IL-6 as a biomarker or treatment target in patients with gastric, bile duct, pancreatic and colorectal cancer. Oncotarget.

[24] Long T.M., Raufman J.-P. (2011). The diagnostic and prognostic role of cytokines in colon cancer. Gastrointest. Cancer Targets Ther..

[25] Park E.Y., Park E., Jin T., Lim M.K., Oh J.K. (2023). Association between Proinflammatory Cytokines and Lung Cancer Risk: A Case-Cohort Study from a Community-Based Prospective Cohort. Cancers.

[26] Baek S., Park E., Park E.Y. (2025). Association of Pro-inflammatory Cytokines with Gastric Cancer Risk: A Case-Cohort Study. Cancer Res. Treat..

[27] Song M., Sasazuki S., Camargo M.C., Shimazu T., Charvat H., Yamaji T., Sawada N., Kemp T.J., Pfeiffer R.M., Hildesheim A. (2018). Circulating inflammatory markers and colorectal cancer risk: A prospective case-cohort study in Japan. Int. J. Cancer.

[28] Wu J., Cai Q., Li H., Cai H., Gao J., Yang G., Zheng W., Xiang Y.B., Shu X.O. (2013). Circulating C-reactive protein and colorectal cancer risk: A report from the Shanghai Men’s Health Study. Carcinogenesis.

[29] Kakourou A., Koutsioumpa C., Lopez D.S., Hoffman-Bolton J., Bradwin G., Rifai N., Helzlsouer K.J., Platz E.A., Tsilidis K.K. (2015). Interleukin-6 and risk of colorectal cancer: Results from the CLUE II cohort and a meta-analysis of prospective studies. Cancer Causes Control.

[30] Bhat A.A., Nisar S., Singh M., Ashraf B., Masoodi T., Prasad C.P., Sharma A., Maacha S., Karedath T., Hashem S. (2022). Cytokine- and chemokine-induced inflammatory colorectal tumor microenvironment: Emerging avenue for targeted therapy. Cancer Commun..

[31] Zaidi M.R., Merlino G. (2011). The two faces of interferon-γ in cancer. Clin. Cancer Res..

[32] Wang L., Wang Y., Song Z., Chu J., Qu X. (2015). Deficiency of interferon-gamma or its receptor promotes colorectal cancer development. J. Interferon Cytokine Res..

[33] Zuo H., Tell G.S., Vollset S.E., Ueland P.M., Nygård O., Midttun Ø., Meyer K., Ulvik A., Eussen S.J. (2014). Interferon-γ-induced inflammatory markers and the risk of cancer: The Hordaland Health Study. Cancer.

[34] Liu X. (2025). The paradoxical role of IFN-γ in cancer: Balancing immune activation and immune evasion. Pathol. Res. Pract..

[35] Schleh M.W., Caslin H.L., Garcia J.N., Mashayekhi M., Srivastava G., Bradley A.B., Hasty A.H. (2023). Metaflammation in obesity and its therapeutic targeting. Sci. Transl. Med..

[36] Johnson I.T., Lund E.K. (2007). Review article: Nutrition, obesity and colorectal cancer. Aliment. Pharmacol. Ther..

[37] John B.J., Irukulla S., Abulafi A.M., Kumar D., Mendall M.A. (2006). Systematic review: Adipose tissue, obesity and gastrointestinal diseases. Aliment. Pharmacol. Ther..

[38] Festa A., D’Agostino R., Williams K., Karter A.J., Mayer-Davis E.J., Tracy R.P., Haffner S.M. (2001). The relation of body fat mass and distribution to markers of chronic inflammation. Int. J. Obes. Relat. Metab. Disord..

[39] Park H.S., Park J.Y., Yu R. (2005). Relationship of obesity and visceral adiposity with serum concentrations of CRP, TNF-alpha and IL-6. Diabetes Res. Clin. Pract..

[40] Osório-Costa F., Carvalheira J.B. (2013). TNF-α in obesity-associated colon cancer. Transl. Gastrointest. Cancer.

[41] Carlini V., Noonan D.M., Abdalalem E., Goletti D., Sansone C., Calabrone L., Albini A. (2023). The multifaceted nature of IL-10: Regulation, role in immunological homeostasis and its relevance to cancer, COVID-19 and post-COVID conditions. Front. Immunol..

[42] Esposito K., Pontillo A., Giugliano F., Giugliano G., Marfella R., Nicoletti G., Giugliano D. (2003). Association of low interleukin-10 levels with the metabolic syndrome in obese women. J. Clin. Endocrinol. Metab..

[43] Engeli S., Janke J., Gorzelniak K., Böhnke J., Ghose N., Lindschau C., Luft F.C., Sharma A.M. (2004). Regulation of the nitric oxide system in human adipose tissue. J. Lipid Res..

[44] Mandal P. (2018). Molecular signature of nitric oxide on major cancer hallmarks of colorectal carcinoma. Inflammopharmacology.

[45] PeÑarando J., Aranda E., RodrÍguez-Ariza A. (2019). Immunomodulatory roles of nitric oxide in cancer: Tumor microenvironment says “NO” to antitumor immune response. Transl. Res..

[46] Mandal P. (2018). Insight of nitric oxide signaling: A potential biomarker with multifaceted complex mechanism in colorectal carcinogenesis. Biochem. Biophys. Res. Commun..

[47] Catalano T., Selvaggi F., Cotellese R., Aceto G.M. (2025). The Role of Reactive Oxygen Species in Colorectal Cancer Initiation and Progression: Perspectives on Theranostic Approaches. Cancers.

[48] Leufkens A.M., van Duijnhoven F.J., Woudt S.H., Siersema P.D., Jenab M., Jansen E.H., Pischon T., Tjønneland A., Olsen A., Overvad K. (2012). Biomarkers of oxidative stress and risk of developing colorectal cancer: A cohort-nested case-control study in the European Prospective Investigation into Cancer and Nutrition. Am. J. Epidemiol..

[49] Halliwell B., Whiteman M. (2004). Measuring reactive species and oxidative damage in vivo and in cell culture: How should you do it and what do the results mean?. Br. J. Pharmacol..

